# Higher Dietary Cost Is Associated with Higher Diet Quality: A Cross-Sectional Study among Selected Malaysian Adults

**DOI:** 10.3390/nu9091028

**Published:** 2017-09-16

**Authors:** Ibnteesam Pondor, Wan Ying Gan, Geeta Appannah

**Affiliations:** Department of Nutrition and Dietetics, Faculty of Medicine and Health Sciences, Universiti Putra Malaysia, Serdang, Selangor 43300, Malaysia; ibteesampondor@gmail.com (I.P.); wanying@upm.edu.my (W.Y.G.)

**Keywords:** diet quality, food prices, dietary cost, healthy eating index, Malaysian adults

## Abstract

Food price is a determining factor of food choices; however its relationship with diet quality is unclear in Malaysia. This study aimed to examine socio-economic characteristics and daily dietary cost (DDC) in relation to diet quality in the state of Selangor, Malaysia. Dietary intake was assessed using a Food Frequency Questionnaire (FFQ) and diet quality was estimated using a Malaysian Healthy Eating Index (M-HEI). DDC in Malaysian Ringgit (RM) was calculated from dietary intake and national food prices. Linear regression models were fitted to determine associations between DDC and M-HEI scores and predictors of diet quality. The mean M-HEI score of respondents was 61.31 ± 10.88 and energy adjusted DDC was RM10.71/2000 kcal (USD 2.49). The highest quintile of adjusted DDC had higher M-HEI scores for all respondents (Q1: 57.14 ± 10.07 versus Q5: 63.26 ± 11.54, *p* = 0.001). There were also positive associations between DDC and M-HEI scores for fruits (*p* < 0.001) and vegetables (*p* = 0.017) for all respondents. Predictors of diet quality included carbohydrate (*β* = 0290; *p* < 0.001) and fat intakes (*β* = −0.242; *p* < 0.001) and energy adjusted DDC (*β* *=* 0.196; *p* < 0.001). Higher dietary cost is associated with healthy eating among Malaysian adults.

## 1. Introduction

Behavioural economics suggests that, when faced with different options regarding food consumption, individuals will make choices that best serve their own utility, which is, however, driven by financial constraints, lack of time, social and cultural norms as well as health concerns [1]. Food habits of Malaysians as any other diverse population are influenced by socio-economic position, race and culture [2]. Conversely, the availability and acceptability of healthier alternatives are known to deter the promotion of healthy diets especially because of the cost factor [3]. Food price and consequently individual dietary cost largely influence the food environment and is the leading influence on food choices [4]. It is known that foods that are healthy yet aligned with social norms are higher in cost [5]. Reviews based on developed countries have concluded that higher energy adjusted daily dietary cost is positively associated with higher diet quality [3,6,7]. Additionally, better educated and more affluent people would consume higher-quality diets and thus accounting for socioeconomic disparities in diet quality [6,7,8]. Food patterns that are altogether affordable, nutrient dense and appealing should be identified and prioritized to combat this inequality [7].

While only few studies linking dietary cost and diet quality have been carried out in Japan, this research area remains greatly unexplored in Asia [9,10,11]. Generally, this topic has been tackled in high income countries while the same equation remains novel in low and middle-income countries [4,12,13,14]. Despite undergoing rapid economic development and having major improvements in lifestyle and eating patterns, Malaysia has been experiencing increasing rates of obesity from 15.4% in 2011 to 18.5% in 2015 [15,16]. In general, there is a need for improvement in diet quality for the average Malaysian adult [17,18]. Although several measures are being taken by the Ministry of Health, Malaysia, there is a need to investigate multispectral strategies for preventive health. For instance, studies from real-life settings have shown that there is a tendency to reduce consumption of unhealthy foods when the price of the latter increases [19]. Increasing Daily Dietary Cost (which is the total cost of foods consumed per day [14]) was associated with both better weight management and improved diet quality [13]. While the prices of food are ever-increasing in Malaysia and around the world, it is thus important to examine the DDC (Daily Dietary Cost) of the Malaysian population and its relationship to diet quality [20].

## 2. Materials and Methods

### 2.1. Study Participants

This cross-sectional study was conducted among 450 healthy adults aged 18 to 64 years in three districts of the State of Selangor, Malaysia. They did not have any physical or mental disability nor did the the study consist of pregnant women. The study proceeded by a two-stage cluster sampling where *Petaling, Hulu Langat* and *Gombak* were chosen from the nine existing districts. From these selected districts, three towns (one in each district) were randomly designated from the local authority areas. Then, two housing areas were randomly selected from each of the three towns, whereby 450 respondents were recruited proportionately. Data collection took place from April to July 2016 after obtaining ethical approval (Reference No: FPSK (EXP15) P171) from the Ethic Committee for Research Involving Human Subjects Universiti Putra Malaysia. Written informed consent was sought and obtained from the respondents prior to the beginning of the survey.

### 2.2. Assessment of Socio-Economic Characteristics

For the present study, socio-economic characteristics were described as education level, household and personal income, age, occupation classification, marital status and housing type. Education level was categorized into: no formal education, primary school, secondary school, diploma, undergraduate degree, master degree and PhD. Household income in Malaysian Ringgit was grouped into: ≤2299, 2300–5999 and ≥6000 and a range of : <1500, 1500–3500 and >3500 for personal income was used [21]. Age was categorized into 18–19, 20–29, 30–39, 40–49, 50–59 and ≥60 years [22]. Occupations were classified according to the Malaysia Standard Classification of Occupations 2013 [23]. Respondents were also asked about marital status (married, single, widowed and divorced) and housing type (detached, semi-detached, terrace, low-cost and shop lot housing as per type of living quarters in Malaysia [24]).

### 2.3. Dietary Intake

Dietary intake was assessed using a validated semi-quantitative FFQ, which consisted of 126 food items. The food items were collapsed into 13 food groups according to their usage (refer to Supplementary Table S1) [25]. The serving size is based on the Food Portion Sizes of Malaysian Foods Album 2002/2003. Following the conversion of food items into food intake (gram), they were entered into Nutritionist Pro™ software (First DataBank Corporation, Axxya Systems, Stafford, TX, USA) using the Nutrient Composition of Malaysian Foods database and energy, protein, carbohydrate and fat intakes were estimated [26]. They were also energy adjusted for analysis purposes.

### 2.4. Diet Quality

Diet quality of the respondents was assessed using a Malaysian-Healthy Eating Index (M-HEI). This was adapted from a Healthy Eating Index (HEI), which was developed and validated in a Malaysian adult population [27]. It was based on nine components: seven food groups (cereals and grains; vegetables; fruits; meat, poultry and egg; fish and seafood; legumes and dairy products), which measured adherence of diet to the Malaysian Dietary Guidelines 2010, while the two other nutrients measured total fat consumption as a percentage of total caloric intake and total sodium intake [28]. It is to be noted that, due to the use of a pre-defined FFQ, no distinction could be made for refined and unrefined cereals and grains. All of the components have a score ranging from 0 (for lack of compliance) to 10 (for full compliance). M-HEI has a score of 100; therefore, the total score was obtained by summing up the component scores of each of the nine components, and expressing it as a percentage. The overall score and each sub-score are calculated on a per-calorie basis and are therefore energy adjusted. A score approaching 100 is considered as high diet quality while a score closer to zero is considered as low diet quality.

### 2.5. Dietary Cost Calculation

One of the most suitable methods to measure DDC is to make use of FFQ and supermarket prices [29]. The highest correlation (*r*^2^ = 0.66) between energy intake and cost was obtained by this method when compared to diet diaries and food expenditure (*r*^2^ = 0.06) or diet diaries and supermarket prices (*r*^2^ = 0.24) [29]. The current study made use of the national database of price list for August 2016 from the Ministry of Trade, Cooperative and Consumerism, Malaysia to calculate the daily dietary cost of food items. The database consisted of 256 food items and it had two sections whereby there were prices for wet markets as well as supermarkets/hypermarkets in all districts of Selangor. Average prices for the state of Selangor were also included and these were used for the purpose of calculations. For each food item, the price for 100 g was obtained.

DDC was calculated for 120 food items from the FFQ and the total cost of food was obtained by summing them up. Alcoholic beverages were excluded because the latter would influence the cost in a disproportionate manner due to their high-price [30]. In addition, the price elasticity of demand makes alcoholic beverages quite a complex product, which requires a distinct investigation. As for other beverages, plain water was also excluded, as it was not practical to separate tap filtered water from purchased bottled water [30]. Moreover, the price estimation did not account for non-edible portions in the present study. This is due to the nature of the semi-quantitative FFQ and the monetary value of the non-edible part, especially for ready-to-eat items, is not known.

The total DDC was energy adjusted, which is a standard adjustment technique in epidemiological studies [31]. Adjusting for energy allows for fairer comparisons between sub-populations that are to have different energy requirements or intakes. Moreover, it allows for increased comparability to previous studies [32]. It is to be noted that the present study chose to use the density method to adjust for energy per 2000 kcal (also as an average requirement for Malaysian adults). In addition, energy adjusted DDC was categorized into quintiles to demonstrate the distinction between its extremes in relation to diet quality.

### 2.6. Statistical Analysis

All statistical analyses were conducted using IBM SPSS Statistics Version 22.0 (IBM Corp., Armonk, NY, USA). All reported *p*-values were two-tailed and a significance level of *p* < 0.05 was used. Descriptive statistics were used to present socio-economic characteristics, M-HEI scores and DDC. Normality of data was checked by referring to skewness and Kolmogorov–Smirnov statistics. Differences in mean M-HEI scores by DDC quintiles were tested using ANOVA. In the case of statistical significance, Tukey post hoc test was used to determine differences in M-HEI scores within the quintiles of DDC. Additionally, a linear regression model (controlled for age and personal income) was used to determine associations between quintiles of DDC and M-HEI scores for all respondents.

Another linear regression model (controlled for age and personal income) including all component scores of M-HEI was fitted to find associations between component scores of M-HEI with regards to DDC quintiles. Median values of each DDC quintile were obtained and treated as continuous (dependent variable) in the regression model. To determine significant contributors (based on the resulting *β* coefficients and *p*-values of simple linear regression) to diet quality, a multivariate linear regression model (controlled for age) was fitted. Those variables that also showed statistically significant differences in the bivariate analysis were included in the multivariate linear regression model. Supplementary Table S2 indicates those variables included in the multivariate regression model.

## 3. Results

The socio-economic characteristics of respondents, the mean M-HEI, and the crude and the energy adjusted DDC are presented in Table 1. Out of 450 respondents, 35.8% were males and 64.2% were females. Most of the respondents were in the age group of 30–39 years (34.4%) and their mean age was 37.55 ± 11.0 years. The majority of them were working adults (66.0%), lived in owned accommodation (62.0%) and were married (72.2%). More than half of the respondents resided in low cost housing (58.4%). Almost all of the respondents had attained at least secondary school education (97.1%). Some differences were noted within the socio-economic characteristics demarcations with regards to diet quality and DDC. For instance, those in the age group ≥ 60 years had higher mean M-HEI (70.22 ± 11.95) compared to those of 20–29 years old (59.70 ± 9.90; *p* = 0.04). Similarly, the same age group had higher DDC (RM25.05; *p* < 0.04) compared to those in the 18–19 years (RM17.84) but the latter was completely reduced when energy adjustment was carried out.

Cereal products (19.7%), non-alcoholic beverages (18.1%), fruits and vegetables (15.1%) and confectionaries (13.0%) food groups showed an important contribution to the total DDC (Table 2). The protein-rich food groups altogether such as meat and poultry; fish and seafood; eggs; legumes and milk products accounted roughly to 29.3%.

As per Table 3, there was a positive association between higher energy adjusted DDC and higher mean M-HEI scores for total respondents (*p* < 0.001). There were significant positive associations between higher energy adjusted DDC and higher component M-HEI scores for cereal products (*p* = 0.027), vegetables (*p* = 0.017) and fruits (*p* < 0.001) for all respondents. In general, there was an increasing trend in M-HEI score with higher quintiles of energy adjusted DDC for all components except for fat and sodium. In addition, Table 4 demonstrated the predictors of diet quality which included adjusted carbohydrate (*β* = 0.290, *p* < 0.001) followed by adjusted fat (*β* = −0.242, *p* < 0.001) and energy adjusted DDC (*β* = 0.196, *p* < 0.001).

## 4. Discussion

The present study showed a positive association between higher energy adjusted DDC and higher mean M-HEI scores and more specifically for component scores of cereal products, vegetables and fruits. These food groups also formed part of the highest contributors to total DDC of the respondents.

Out of all the socio-economic characteristics surveyed, higher M-HEI scores and higher adjusted DDC/2000 kcal were obtained for older respondents compared to younger ones. It is generally accepted that higher socio-economic position has consistently been associated with better diet quality [6,33] and that they are usually accompanied by better health outcomes [34].

Assigning monetary value to individual-level dietary data using a database of national average food prices is an increasingly popular method that has been previously employed to report monetary value of the diets of French, American, Japanese, Spanish and British adults [4,11,12,13,35]. This is the first time that a monetary value has been incorporated to dietary data in the Malaysian population. The first and second items in the top ten mostly consumed foods by Malaysian adults on a daily basis were white rice (2.48 plates) and white/brown sugar (3.65 teaspoons) [22]. This is therefore in line with data reported in Table 2, whereby cereal products and confectionaries were among the highest contributors to DDC.

Significant positive association obtained between higher energy adjusted DDC and higher mean M-HEI scores for total respondents confirmed findings from the literature [7,12,13] that associated better diet quality with higher monetary cost of diets. Although the current study presented similar results to Western countries, it should be noted that one study involving young Japanese women concluded that higher dietary cost was not automatically associated with healthier dietary pattern [10]. For instance, it was found that higher quintiles of DDC were associated with higher intakes of fruits, vegetables, fish and pulses as well as meat, non-alcoholic beverages and oils [10]. This difference between Western countries and Asian ones can be due to differing social and cultural backgrounds that logically extend to dietary patterns [11].

While this study showed that fruits and vegetables contributed to 15.1% of DDC, the same results were higher for Japanese adults (24.6%) [11]. Fruits and vegetables are known to be relatively more expensive than other food groups and have been a main argument and economic barrier against the adherence to a healthy diet [12,14]. Although higher dietary cost was associated with more healthful diets, one researcher stated that strategies to eat healthily and more affordably should be directed towards the consumption of plant-based foods that ultimately offer the best bargain for dietary health [36]. This is in disagreement with some above-mentioned studies linking higher monetary value of diets and diet quality to the prices of fruits and vegetables. Among 94 foods and beverages in the UK consumer price index, all prices had increased over the years 2002–2012, but healthier food items had increased more rapidly in absolute terms per year compared to less healthy items, respectively [37]. This is a perturbing finding and the trend of healthier diets becoming less affordable over time is being observed altogether with the present study.

As for the predictors of diet quality from the multiple linear regression, it could have been expected that DDC would play a more important role in the model, taking into consideration the previous results of the study. However, the literature recently concluded that socio-economic characteristics acted as a mediator between cost and healthier diets [34,38]. The present study did not find any significant differences in M-HEI score or DDC with regards to socio-economic characteristics with the exception of age groups. Thus, the absence of significant relationships with regards to socio-economic status could have weakened the regression model predicting diet quality. Additionally, the highest contributor to the model was adjusted carbohydrate. The use of a pre-defined FFQ presented a limitation, as it did not distinguish between refined and unrefined cereals in the present study. It is thus possible that the high contribution of carbohydrate to the diet quality represented the consumption of unrefined cereals.

In addition, the use of a FFQ to gauge diet quality could be questioned; for instance, while M-HEI components did capture the adequacy of each food group, excess dietary intake such as carbohydrate and protein was not adequately evaluated except for a total fat component, whereby a percentage >30% was deemed as excessive.

Moreover, DDC data represent an estimate of the actual dietary costs of individuals as opposed to what they really spend. Factors such as food away from home, food purchased at discounted or wholesale prices, wastage and monetary value of non-edible parts of food items have not been taken into consideration. The nature of the semi-quantitative FFQ being used made it complex to estimate DDC especially for ready-to-eat food items. Hence, these presented limitations in the present study. Lastly, the metric (RM/2000 kcal) used for DDC could be questioned, especially because the present study examined the relationship between dietary cost and diet quality, and it was seen that prices of low energy dense foods such as fruits and vegetables tend to be higher per unit of energy (kcal) compared to mass (g) [7].

## 5. Conclusions

Results from this study showed that higher DDC is associated with higher scores of M-HEI among Malaysian adults. This information serves as a stepping stone to more dietary research that could better discern the role of socio-economic position and cost of foods with regards to diet quality. The bottom line remains that healthier diets could become less affordable over time and have, if not already, presented implications in the public health domain, especially in terms of social inequalities.

## Figures and Tables

**Table 1:** Socio-economic characteristics by mean Malaysian-Healthy Eating Index (M-HEI) scores and median Daily Dietary Cost (DDC).

Socio-Economic Characteristics	*n* (%)	Mean M-HEI ± Standard Deviation	Crude Median DDC/ RM (IQR)	Adjusted DDC/ RM/2000 kcal (IQR)
Total Respondents	450 (100.0)	61.31 ± 10.88	16.87 (14.20)	10.71 (4.49)
Male	161 (35.8)	61.03 ± 10.51	16.50 (14.69)	10.63 (4.46)
Female	289 (64.2)	61.43 ± 11.10	17.27 (13.88)	10.83 (4.57)
Ethnicity				
Malay	369 (82.0)	61.13 ± 11.10	16.87 (13.94)	10.67 (4.28)
Chinese	24 (5.3)	63.57 ± 9.75	15.98 (13.35)	11.12 (6.50)
Indian	54 (12.0)	61.46 ± 10.19	19.87 (16.74)	11.88 (5.54)
Others	3 (0.7)	58.52 ± 3.39	6.78	8.87
Age Classification *				
18–19 years	16 (3.6)	58.26 ± 9.58	17.84 (10.12) ^‡^	10.71 (5.64)
20–29 years	95 (21.1)	59.70 ± 9.90 ^†^	15.97 (11.23)	10.46 (3.74)
30–39 years	155 (34.4)	62.27 ± 11.02	18.68 (16.76)	11.08 (5.43)
40–49 years	104 (23.1)	61.12 ± 11.27	15.62 (14.16)	10.63 (3.63)
50–59 years	70 (15.6)	60.92 ± 10.88	16.35 (12.05)	10.78 (4.74)
≥60 years	10 (2.2)	70.22 ± 11.95 ^†^	25.05 (21.97) ^‡^	11.52 (6.01)
Education Level				
No formal education	2 (0.4)	56.67 ± 0.00	13.65	10.78
Primary school	11 (2.4)	59.72 ± 14.91	17.24 (26.93)	9.51 (3.78)
Secondary school	204 (45.3)	61.12 ± 10.75	17.13 (14.53)	10.75 (4.75)
Diploma	103 (22.9)	61.06 ± 11.00	16.25 (13.80)	10.62 (4.45)
Bachelor’s degree	107 (23.8)	61.96 ± 10.71	17.27 (13.86)	10.71 (4.40)
Master’s degree	17 (3.8)	60.65 ± 10.91	15.09 (16.03)	10.75 (3.45)
PhD	6 (1.3)	65.56 ± 12.47	25.42 (28.75)	13.74 (9.15)
Housing				
Owned	279 (62.0)	61.77 ± 11.41	16.86 (13.59)	10.88 (4.29)
Rented	171 (38.0)	60.46 ± 9.92	17.04 (14.88)	10.53 (5.30)
Housing type				
Detached	10 (2.2)	56.56 ± 8.39	17.74 (15.18)	
Semi-detached	22 (4.9)	65.86 ± 9.78	17.81 (8.84)	11.13 (7.30)
Terrace	141 (31.3)	61.31 ± 11.19	17.13 (14.07)	11.36 (2.99)
Low-cost	268 (59.6)	61.09 ± 10.73	16.90 (14.36)	10.90 (4.72)
Shop Lot	9 (2.0)	60.74 ± 14.16	10.90 (24.87)	10.29 (4.30)
Personal income *				
<RM1500	54 (12.0)	58.87 ± 11.93	16.02 (16.33)	8.80 (3.67)
RM 1500–3500	175 (38.9)	60.66 ± 10.81	17.69 (15.15)	10.00 (3.92)
>RM3500	112 (24.9)	62.34 ± 10.08	16.02 (13.12)	10.84 (4.56)
Household income *				10.74 (4.53)
≤RM2299	85 (18.9)	60.71 ± 11.96	16.53 (14.14)	
RM2300–5999	223 (49.6)	60.56 ± 11.01	17.32 (14.45)	9.73 (4.44)
≥RM6000	131 (29.1)	62.65 ± 10.46	16.94 (13.09)	10.73 (4.45)
Marital Status				11.06 (4.43)
Single	109 (24.2)	59.77 ± 10.25	16.46 (13.23)	
Married	325 (72.2)	61.60 ± 11.08	17.17 (14.47)	10.19 (4.45)
Widow	8 (1.8)	63.33 ± 10.67	14.31 (11.65)	10.75 (4.48)
Divorced	8 (1.8)	67.08 ± 9.78	19.73 (17.69)	9.33 (6.12)
Occupation **				10.77 (7.97)
Managers	21 (4.7)	63.54 ± 11.86	18.93 (12.83)	
Professionals	102 (22.7)	62.45 ± 10.07	16.49 (15.77)	10.92 (4.16)
Technicians	47 (10.5)	58.75 ± 12.01	15.13 (14.92)	10.63 (4.89)
Clerical Support	48 (10.7)	61.90 ± 9.06	18.88 (16.35)	9.82 (4.58)
Service & Sales	49 (10.9)	59.25 ± 9.94	17.85 (14.49)	11.04 (4.97)
Craft and Trade	11 (2.4)	56.36 ± 8.38	16.50 (6.00)	11.00 (4.11)
Machine Operators	11 (2.4)	56.97 ± 13.88	12.14 (13.20)	11.04 (5.86)
Pensioner/ Unemployed Students	145 (32.2)	62.07 ± 11.11	17.76 (13.03)	9.77 (3.35)

DDC was presented in median and interquartile range (IQR) in Malaysian Ringgit (RM). * Age classification as per the Malaysian National Health and Morbidity Survey [22] and Household/Personal income range as per The Malaysian Economic Planning Unit [21]. ** Occupation Classification according to Malaysian Standard Classification of Occupations [23]. ^†^ Tukey post hoc test revealed a significant difference in M-HEI score (*p* = 0.04). ^‡^ Tukey post hoc test revealed a significant difference in crude DDC (*p* = 0.04).

**Table 2:** Percentage of food group intakes contributing to total DDC.

Food Groups *	Mean % Contribution to Total DDC	Standard Deviation
Cereal and cereal products (17)	19.7	11.20
Non-alcoholic beverages (10)	18.1	10.29
Fruits and vegetables (30)	15.1	10.00
Confectionaries (8)	13.0	11.95
Meat and meat products (12)	9.1	7.51
Fish and fish products (12)	8.3	6.60
Milk and milk products (6)	7.0	8.08
Condiments (11)	3.3	3.31
Eggs (4)	2.6	2.89
Legumes and products (4)	2.2	2.76
Spreads (6)	1.6	2.32

* 120 items are used in DDC calculation, these are divided into 11 food groups, each having the above listed number of items.

**Table 3:** Relationships between mean M-HEI component scores and energy-adjusted DDC quintiles.

M-HEI Components	Total Respondents (*N* = 450)					
	Quintile 1 * (≤RM8.32) (*n* = 93)	Quintile 2 (RM8.33–9.77) (*n* = 84)	Quintile 3 (RM9.78–11.37) (*n* = 99)	Quintile 4 (RM11.38–13.92) (*n* = 95)	Quintile 5 (≥RM13.93) (*n* = 79)	*p*-Value
M-HEI Score	57.14 ± 10.07	61.14 ± 9.60	61.86 ± 10.00	63.02 ± 12.09	63.26 ± 11.54	0.001 ^†^
Cereals	9.95 ± 0.52	10.00 ± 0.00	10.00 ± 0.00	10.00 ± 0.00	10.00 ± 0.00	0.027
Poultry & Meat	5.09 ± 3.74	5.58 ± 3.27	5.75 ± 3.48	7.27 ± 3.35 ^c^	8.11 ± 3.13 ^c^	0.268
Fish & Seafood	5.44 ± 3.77	6.00 ± 3.45	7.14 ± 3.20 ^b^	8.05 ± 3.15 ^c^	9.62 ± 1.33 ^c^	0.331
Legumes	2.28 ± 3.32 ^c^	4.60 ± 4.01 ^c^	6.47 ± 3.96 ^c^	7.05 ± 3.99 ^c^	8.44 ± 2.95 ^c^	0.297
Milk Products	3.12 ± 2.92	3.55 ± 2.66	3.77 ± 2.86	5.35 ± 3.35 ^c^	6.96 ± 3.12 ^c^	0.668
Vegetables	4.14 ± 2.24	4.42 ± 2.72	5.18 ± 2.79	6.93 ± 2.98 ^c^	8.52 ± 2.52 ^c^	0.017
Fruits	3.87 ± 2.73 ^a^	5.04 ± 2.88 ^c^	5.79 ± 2.94 ^c^	7.72 ± 2.94 ^c^	9.38 ± 1.76 ^c^	<0.001
Fat **	6.24 ± 4.15	6.85 ± 4.09	7.47 ± 3.38	6.16 ± 4.22	7.41 ± 3.57	0.971
Sodium **	1.99 ± 3.91	3.69 ± 4.67	3.33 ± 4.57	2.16 ± 4.04	1.52 ± 3.52	0.130

Quintile 1 *: Reference. ** Higher scores represent lower consumption of saturated fat and sodium. Significance of pairwise differences (ANOVA/Tukey post hoc) compared with the reference group: ^a^ *p* < 0.05, ^b^ *p*< 0.01, ^c^ *p* < 0.001. ^†^ *p*-value obtained from a linear regression model adjusted for age and personal income for M-HEI score and DDC quintiles (Total respondents *R*^2^ = 0.046; *df* (5,444) = 4.24; *p* = 0.001). *p*-value obtained from a linear regression model adjusted for age and personal income that treated diet cost quintiles as continuous by modelling the median value of each quintile category (dependent variable) and all M-HEI component scores (Total respondents *R*^2^ = 0.094; *df* (10,439) = 4.53; *p* < 0.001).

**Table 4:** Determinants of diet quality from a multiple linear regression model.

Variables	Unstandardized B	95% Confidence Interval for B		*β* Coefficients	*p*-Value
Adjusted protein (% kcal)	0.056	−0.026	0.136	0.084	0.106
Adjusted CHO (% kcal)	0.082	0.049	0.115	0.290	<0.001
Adjusted fat (% kcal)	−0.086	−0.135	−0.034	−0.242	<0.001
Adjusted DDC (RM/2000 kcal)	0.226	0.108	0.338	0.196	<0.001

*R*^2^ = 0.178. *F* (7, 442) = 13.66. *p* < 0.001: Controlled for age.

## Supplementary Materials

The following are available online at http://www.mdpi.com/2072-6643/9/9/1028/s1, Table S1: Food groups and their food items and Table S2: Correlation between adjusted and unadjusted nutrient intakes, DDC and M-HEI.
