# Supplementary material for: Higher Dietary Cost Is Associated with Higher Diet Quality: A Cross-Sectional Study among Selected Malaysian Adults
Source: Nutrients. 2017 Sep 16;9(9):1028. doi: 10.3390/nu9091028 (PMC5622788; doi:10.3390/nu9091028)
Supplement: Supplementary file 1 [file nutrients-09-01028-s001.docx]

Supplementary Table 1. Food groups and their food items [24]

| **Food groups** | **Food items** |
| --- | --- |
| Cereals and cereal products | Rice, Porridge, Sticky rice, Yellow noodles, *Mihun/ Kueh Teow*, Loh Shi Fun, Pasta, Sago, Bread, Bread bun, Indian bread, Indian flatbread, South-Indian pancake, Processed Breakfast cereals, Uncooked cereals, Pizza, Corn |
| Meat and meat products | Chicken, Beef, Lamb, Meat burger, Hotdog/ Sausage, Nugget, Chicken ball, Duck, Ham, Bacon, Luncheon meat, Pork |
| Fish and seafood | Salt water fish, Fresh water fish, Anchovy, Tinned fish, Shellfish, Fresh shrimp, Fresh cuttlefish, Dried cuttlefish, Crab, Salted fish, Fish ball, Fish Crackers |
| Eggs | Chicken egg, Duck egg, Quail egg, Salted egg |
| Beans and bean products | Beans, Tofu, Tempeh, Peanuts |
| Milk and milk products | Fresh milk, Powdered milk, Evaporated milk, Condensed milk, Yogurt, Cheese |
| Vegetables | Greens, Peas, Starchy vegetables, Cabbage, Squash, Salted vegetables, *Ulam-ulam*, Corn bud, Mushroom, Beansprout |
| Fruits | Papaya, Guava, Orange, Mango, Pineapple, Banana, Watermelon, Starfruit, Jackfruit, Apple, Mandarine, Pear, Grapes, Durian, Rambutan, Fresh longan, Litchi, Honeydew, Tinned fruits, Dried fruits |
| Non-alcoholic Drinks | Plain water, Tea, Coffee, Chocolate drinks, Malted drinks, Cordial syrup, Fruit juice, Soft drinks, Soya drink, Herbal drinks, Isotonic drinks |
| Alcoholic drinks | Shandy, Beer, Spirit, Liqueur, Wine |
| Confectionaries | Local Cakes, Pastries, Biscuits, Candy, Ice cream, ABC (*Air Batu Campur*), Custard sauce/jellies, Crackers |
| Spreads | Jam, Coconut jam, Butter, Margarine, Peanut butter, Cream cheese |
| Condiments | Sugar, Honey, *Sambal belacan*, Fermented anchovy, Fermented shrimp, Heavy soy sauce, Light soy sauce, Chili/ tomato sauce, Oyster sauce, Fish sauce, Shrimp sauce |

Supplementary Table 2. Correlation between adjusted and unadjusted nutrient intakes, DDC and HEI

|  | **Unadjusted** | | | **Energy Adjusted** | | |
| --- | --- | --- | --- | --- | --- | --- |
|  | **Variables** | **r** | ***p*-value** | **Variables** | **r** | ***p*-value** |
| **HEI Score** | Carbohydrate (kcal) | 0.400 | < 0.001 | Carbohydrate (% kcal) | 0.197 | < 0.001 |
|  | Protein (kcal) | 0.348 | < 0.001 | Protein (% kcal) | 0.156 | < 0.001 |
|  | Fat (kcal) | 0.300 | < 0.001 | Fat (% kcal) | 0.131 | 0.005 |
|  | DDC (RM) | 0.298 | < 0.001 | DDC (RM/2000kcal) | 0.201 | < 0.001 |
